# Gallstone disease and nonalcoholic fatty liver disease in patients with type 2 diabetes: a cross-sectional study

**DOI:** 10.1186/s12902-021-00899-z

**Published:** 2021-11-19

**Authors:** Ye Lu, Lili Hu, Jing Song, Jing Wan, Haibing Chen, Jun Yin

**Affiliations:** 1 Department of Endocrinology and Metabolism, Shanghai Eighth People’s Hospital, Shanghai, 200235 China; 2 Department of Endocrinology and Metabolism, Shanghai Jiao Tong University Affiliated Sixth People’s Hospital, Shanghai Clinical Center for Metabolic Diseases, Shanghai Key Laboratory of Diabetes Mellitus, Shanghai Diabetes Institute, Shanghai, 200233 China; 3 Department of Endocrinology and Metabolism, Shanghai 10th People’s Hospital, Tongji University, Shanghai, 200072 China

**Keywords:** Type 2 diabetes mellitus, Gallstone disease, Cholecystectomy, Nonalcoholic fatty liver disease

## Abstract

**Background:**

Nonalcoholic fatty liver disease (NAFLD) and gallstone disease (GSD) often coexist in the general population owing to shared risk factors. This study explored the relationship between NAFLD and GSD in patients with type 2 diabetes.

**Methods:**

We conducted a retrospective cross-sectional analysis of 4325 patients with type 2 diabetes. GSD and NAFLD were confirmed using ultrasonography. GSD was defined as either asymptomatic gallstones or previous cholecystectomy, and each was analyzed separately.

**Result:**

There was no significant difference in the prevalence of GSD between patients with and without NAFLD (23.8% vs. 21.2%, *P* = 0.15). After case–control matching (1:1) of baseline data such as age, sex, duration of diabetes, and HbA1c between patients with and without NAFLD, there was still no significant difference in the prevalence of GSD (25.5% vs. 23.6%, *P* = 0.15). The prevalence of NAFLD in patients with asymptomatic gallstones was lower than that of patients without GSD (38.6% vs. 47.3%, *P* < 0.001), whereas the prevalence in those who had undergone cholecystectomy was much higher (61.2% vs. 47.3%, *P* < 0.001). The ratio of cholecystectomy to asymptomatic gallstone in patients with or without NAFLD was 1.97 and 0.79, respectively. The rate of cholecystectomy was higher in the patients with NAFLD than in those without NAFLD (15.8% vs. 9.3%, *P* < 0.001), consistent with the result after case–control matching (17.3% vs. 11.2%, *P* < 0.001). Multivariate logistic regression analysis, after adjusting for numerous potential confounding factors, revealed that GSD (OR = 1.241, 95%CI: 1.036–1.488, *P* = 0.002) and cholecystectomy (OR = 1.946, 95%CI: 1.546–2.445, *P* < 0.001) were both strongly associated with NAFLD. However, asymptomatic gallstone (OR = 0.663, 95%CI: 0.513–0.856, *P* = 0.002) seemed to be negatively correlated with NAFLD.

**Conclusions:**

The prevalence of GSD was similar in patients with type 2 diabetes with and without NAFLD. The higher proportion of cholecystectomy and lower proportion of asymptomatic gallstones in patients with NAFLD suggests that NAFLD may increase the risk of complications of GSD.

## Background

The liver is an essential organ in systemic metabolism, contributing substantially to the development of insulin resistance and type 2 diabetes mellitus (T2DM). Nonalcoholic fatty liver disease (NAFLD) refers to histological liver abnormalities ranging from isolated steatosis to nonalcoholic steatohepatitis (NASH), which may result in cirrhosis and is related to being overweight or obese and having metabolic disorders, such as insulin resistance, T2DM, and dyslipidemia [1]. NAFLD and T2DM often coexist and appear to synergistically increase the risk of hepatic and extrahepatic clinical outcomes [2, 3]. T2DM worsens the course of NAFLD, which in turn makes diabetes treatment more challenging [4].

Gallstone disease (GSD) is another common condition that exhibits a concomitant increase in prevalence as the incidence of obesity increases [5]. GSD is also positively associated with diabetes risk [6]. In the general population, both GSD and NAFLD are prevalent, and they possess overlapping risk factors, such as obesity and insulin resistance. However, a consequential relationship between NAFLD and GSD has not been confirmed [7]. Nevertheless, several studies have suggested an independent association between the two [8, 9]. Moreover, studies have revealed a relationship between cholecystectomy and NAFLD but no relationship between gallstones and NAFLD, indicating that cholecystectomy promotes NAFLD [10–12]. Therefore, GSD is believed to be independently associated with NAFLD. Moreover, in the general population, cholecystectomy but not asymptomatic cholelithiasis aggravates NAFLD. However, the relationship between GSD (including gallstones and cholecystectomy) and NAFLD in people with T2DM has not been investigated yet. To address this issue, we performed a retrospective cross-sectional study in subjects with T2DM.

## Methods

### Participants

This study included inpatients with T2DM at the Department of Endocrinology and Metabolism of Shanghai Jiao Tong University Affiliated Sixth People’s Hospital between January 2011 and December 2014. Participants suffering from the following conditions were excluded: diabetic ketosis, hyperglycemic hyperosmolar syndrome, and other acute complications of diabetes, hyperthyroidism, acute pancreatitis, acute hepatitis, intrahepatic and intrahepatic bile duct stones, gallbladder polyps, cirrhosis, malignant tumors, and pregnancy. A total of 4325 patients with T2DM (2411 men and 1914 women) were enrolled.

### Definitions of GSD and NAFLD

GSD was confirmed with abdominal ultrasonographic examination, which was performed using a 3.5-MHz ultrasound transducer; the presence of gallstones or the absence of the gallbladder ascribed to previous cholecystectomy was determined. The presence of intraluminal echoes dependent on gravity or attenuation of ultrasonic transmission (acoustic shadowing) was considered diagnostic for gallstones.

NAFLD was defined based on the revised definition proposed by the Chinese Hepatology Association in 2010 [13]: ① No history of alcohol consumption or alcohol equivalent amount < 140 g/week (female < 70 g/week). ② Viral hepatitis, drug-induced liver disease, total parenteral nutrition, Wilson’s disease, autoimmune liver disease and other specific diseases that can lead to fatty liver were excluded. ③ The histological changes of liver biopsy met the pathological diagnostic criteria of fatty liver disease. In view of the difficulty in obtaining histological diagnosis of liver, NAFLD was also defined as liver imaging findings meeting the diagnostic criteria for diffuse fatty liver with no other explanation. The imaging diagnosis of NAFLD was based on abdominal ultrasonography of liver brightness and presence of diffuse echogenicity of the liver parenchyma [14].

### Measurements of clinical parameters

All these data were retrieved from patients’ medical records, including body mass index (BMI), waist circumference (WC), blood pressure (BP) and other clinical characteristics.

The general biochemical parameters, including serum levels of fasting plasma glucose (FPG), triglyceride (TG), total cholesterol (TC), high-density lipoprotein cholesterol (HDL-C), low-density lipoprotein cholesterol (LDL-C), glycosylated hemoglobin (HbA1c), glycosylated albumin (GA), gamma-glutamyl transpeptidase (GGT), alanine aminotransferase (ALT), and aspartate aminotransferase (AST), were determined using an automated biochemical detector (Modular E170, Roche, Tokyo, Japan). Blood samples were collected through venipuncture for laboratory testing from each study participant after overnight fasting. All patients underwent ultrasonographic examination.

### Statistical analysis

SPSS v22.0 (IBM Corp, Armonk, NY) was used for statistical analysis. Continuous variables are presented as mean ± standard deviation, and binary variables as numbers (percentages). The Kolmogorov-Smirnov test was taken to assess the normal distribution of the continuous variables. All continuous variables between cases and controls were compared by the Student’s *t* test, or paired *t* test after a 1:1 case control. Chi-square test was constructed to compare differences for categorical variables. Logistic regression analysis was performed to assess the association between GSD and NAFLD. A *P*-value < 0.05 indicates a statistically significant difference.

## Results

**Fig. 1:**
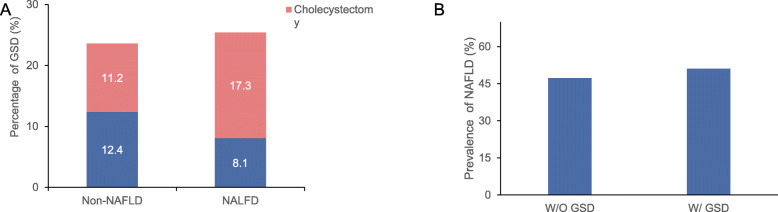
GSD with or without NAFLD and NAFLD with or without GSD in patients with T2DM. a) Prevalence of gallstones or cholecystectomy in patients with and without NALFD. Asymptomatic gallstones were far lower and cholecystectomy much higher in patients with NALFD than in those without NAFLD (*P* < 0.001). b) Prevalence of NAFLD in patients with or without GSD. No significant difference was observed (*P* = 0.15)

The characteristics of the patients with and without NAFLD are presented in Table 1. Compared with patients without NAFLD (*n* = 2241), those with NAFLD (*n* = 2084) were younger (*P* < 0.001) and had higher WC (*P* < 0.001), BMI (*P* < 0.001), and TG (*P* < 0.001). There was no significant difference in the prevalence of GSD between patients with and without NAFLD (23.8% vs. 21.2%, *P* = 0.15). However, the prevalence of gallstones in persons without NAFLD (11.9%) was nearly 1.5 times that in persons with NAFLD (8%, *P* < 0.001). Meanwhile, the prevalence of cholecystectomy was significantly higher in patients with NAFLD (15.8% vs. 9.3%, *P* < 0.001, Fig. 1A). The ratio of cholecystectomy to asymptomatic gallstones in the absence and presence of NAFLD was 0.79 and 1.97, respectively, indicating that the prevalence of cholecystectomy was much higher in patients with NAFLD than in those without NAFLD, whereas there was an opposite trend regarding asymptomatic gallstones (Table 1).

**Table 1:** Characteristics of patients with and without NAFLD

	w/o NAFLD (*n* = 2241)	w/ NAFLD (*n* = 2084)	*P* value
Sex			0.003
Male	1297 (57.9)	1114 (53.4)	
Female	944 (42.1)	970 (46.6)	
Age (years)	61.86 ± 11.57	57.50 ± 12.07	< 0.001
Diabetes duration (years)	11.38 ± 7.27	9.27 ± 6.58	0.001
BMI (kg/m^2^)	23.67 ± 3.18	26.63 ± 3.34	0.012
Waist circumference (cm)			
Male	87.92 ± 9.17	97.01 ± 9.37	< 0.001
Female	86.10 ± 10.32	93.57 ± 9.85	< 0.001
SBP (mmHg)	133.02 ± 17.53	133.29 ± 16.55	0.005
DBP (mmHg)	78.65 ± 9.48	81.24 ± 9.33	0.001
ALT (IU/L)	20.39 ± 24.11	29.36 ± 23.40	< 0.001
AST (IU/L)	20.39 ± 24.11	23.50 ± 15.49	< 0.001
Gamma-GT (IU/L)	30.24 ± 39.20	40.75 ± 40.73	< 0.001
TC (mmol/L)	4.60 ± 1.10	4.85 ± 1.15	< 0.001
TG (mmol/L)	1.29 ± 1.07	2.23 ± 1.99	< 0.001
LDL-C (mmol/L)	2.76 ± 0.87	2.84 ± 0.86	0.003
GA (%)	22.75 ± 7.48	21.14 ± 6.02	< 0.001
HbA1c (%)	8.46 ± 2.18	8.65 ± 1.87	0.003
Gallstone disease	475 (21.2)	496 (23.8)	0.15
Cholecystectomy	209 (9.3)	329 (15.8)	< 0.001
Gallstones	266 (11.9)	167 (8)	< 0.001

Data are presented as the mean ± SD. Binary variables are presented as the number (percentage)

NAFLD, nonalcoholic fatty liver disease; w/o, without; w/, with; BMI, body mass index; SBP, systolic blood pressure; DBP, diastolic blood pressure; ALT, alanine aminotransferase; AST, aspartate aminotransferase; Gamma-GT, gamma-glutamyl transpeptidase; TC, total cholesterol; TG, triglycerides; LDL-C, low-density lipoprotein cholesterol; GA, glycosylated albumin; HbA1c, glycosylated hemoglobin

A 1:1 case–control matching was conducted between patients with and without NAFLD to control for potential confounding factors. After matching for age, sex, HbA1c, and diabetes duration, the prevalence of GSD in patients with and without NAFLD was not significantly different (25.5% vs. 23.6%, *P* = 0.15). However, patients with NAFLD were characterized by higher level of ALT, AST, Gamma-GT as well as TG. Meanwhile, the proportion of patients with NAFLD who underwent cholecystectomy was approximately 1.5 times that of those without NAFLD (17.3% vs. 11.2%; *P* < 0.001). Conversely, the proportion of patients with NAFLD who had asymptomatic gallstones was much lower than those without NAFLD and concomitant asymptomatic gallstones (8.1% vs. 12.4%, *P* < 0.001), which is consistent with the original results (Table 2).

**Table 2:** Characteristics of patients with and without NAFLD after 1:1 case–control matching

	without NAFLD (*n* = 588)	with NAFLD (n = 588)	*P* value
Sex			
Male	331 (56.3%)	331 (56.3%)	
Female	257 (43.7%)	257 (43.7%)	
Age (years)	60.12 ± 9.18	60.08 ± 9.11	0.93
Diabetes duration (years)	10.25 ± 5.36	10.14 ± 5.33	0.74
Waist circumference (cm)			
Male	93.45 ± 6.51	93.71 ± 6.50	0.61.
Female	89.79 ± 7.48	89.87 ± 7.35	0.90
HbA1c (%)	8.11 ± 1.56	8.13 ± 1.46	0.76
ALT (IU/L)	21.61 ± 34.60	26.12 ± 16.68	0.005
AST (IU/L)	19.65 ± 18.70	21.46 ± 9.19	0.035
Gamma-GT (IU/L)	30.11 ± 34.75	36.30 ± 28.91	0.001
TC (mmol/L)	4.60 ± 1.01	4.68 ± 1.10	0.228
TG (mmol/L)	1.39 ± 0.82	2.07 ± 1.34	< 0.001
LDL-C (mmol/L)	2.81 ± 0.84	2.75 ± 0.89	0.307
Gallstone disease	139 (23.6%)	150 (25.5%)	0.15
Cholecystectomy	66 (11.2%)	102 (17.3%)	< 0.001
Gallstones	73 (12.4%)	48 (8.1%)	< 0.001

Data are presented as mean ± SD or number (percentage).

NAFLD, nonalcoholic fatty liver disease; HbA1c, glycosylated hemoglobin

The 4325 patients were assigned to three groups based on GSD status: gallstones (216 men, 217 women), cholecystectomy (204 men, 334 women), and without GSD (1994 men, 1360 women). The prevalence of GSD was 22.45% (971/4325). Compared with patients without GSD, patients with GSD had significantly higher age, female sex, longer diabetes duration, higher BMI, larger WC, higher blood pressure, and higher GGT levels. Regardless of the presence or absence of GSD, the prevalence of NAFLD was similar (51.1% vs. 47.3%, *P* = 0.49; Fig. 1B). However, among the three groups, the prevalence of NAFLD was the highest in patients who underwent cholecystectomy and the lowest in those with asymptomatic gallstones (Table 3).

**Table 3:** Characteristics of patients according to gallstone disease status

	w/o GSD (***n*** = 3354)	Gallstone disease (***n*** = 971)	Gallstone disease (***n*** = 971)	
			Gallstones (***n*** = 433)	Cholecystectomy (***n*** = 538)
Sex				
Male	1994 (59.5)	420 (43.3)***	216 (49.8)***	204 (37.9)***^###^
Female	1360 (40.5)	551 (56.7)	217 (50.2)	334 (62.1)
Age (years)	58.55 ± 12.09	63.87 ± 10.80***	63.92 ± 11.25***	63.92 ± 10.53***
Diabetes duration (years)	10.18 ± 6.92	11.41 ± 7.14***	12.04 ± 6.98**	11.50 ± 7.07***
BMI (kg/m^2^)	25.04 ± 3.56	25.33 ± 3.78*	25.12 ± 3.75*	25.50 ± 3.80*
Waist circumference (cm)				
Male	92.09 ± 10.33	92.38 ± 10.20	92.10 ± 9.79	92.67 ± 10.63
Female	89.33 ± 10.75	91.29 ± 10.65***	90.93 ± 11.33	91.53 ± 10.20
SBP (mmHg)	133.96 ± 16.99	134.91 ± 17.17***	135.15 ± 17.15***	134.72 ± 16.97***
DBP (mmHg)	79.88 ± 9.48	79.94 ± 9.63***	79.68 ± 10.00	80.14 ± 9.32
ALT (IU/L)	24.74 ± 24.11	29.36 ± 23.40	23.34 ± 24.97	25.80 ± 37.47
AST (IU/L)	21.27 ± 24.11	22.34 ± 18.18	21.44 ± 14.40	23.07 ± 21.72
Gamma-GT (IU/L)	34.64 ± 36.15	37.89 ± 52.87*	39.31 ± 54.56*	36.74 ± 51.07
TC (mmol/L)	4.73 ± 1.20	4.68 ± 1.10	4.71 ± 1.10	4.66 ± 1.12
TG (mmol/L)	1.74 ± 1.72	1.77 ± 1.37	1.71 ± 1.19	1.87 ± 1.49^#^
LDL-C (mmol/L)	2.82 ± 0.87	2.73 ± 0.84*	2.79 ± 0.87**	2.69 ± 0.82***
GA (%)	21.74 ± 6.89	21.45 ± 6.68	21.84 ± 6.71	21.16 ± 6.66
HbA1c (%)	8.58 ± 2.06	8.46 ± 1.97	8.52 ± 1.99	8.42 ± 1.96
NAFLD	1588 (47.3)	496 (51.1)	167 (38.6)**	329 (61.2)***^,###^

Data are presented as mean ± SD. Binary variables are presented as the number (percentage)

^*^P < 0.05, ^**^P < 0.01, ^***^P < 0.001, control vs. gallstone disease/ gallstone/ cholecystectomy; ^*#*^*P* < 0.05, ^*##*^*P* < 0.01, ^*###*^*P* < 0.001, gallstone vs. cholecystectomy

NAFLD, nonalcoholic fatty liver disease; w/o, without; w/, with; BMI, body mass index; SBP, systolic blood pressure; DBP, diastolic blood pressure; ALT, alanine aminotransferase; AST, aspartate aminotransferase; Gamma-GT, gamma-glutamyl transpeptidase; TC, total cholesterol; TG, triglycerides; LDL-C, low-density lipoprotein cholesterol; GA, glycosylated albumin; HbA1c, glycosylated hemoglobin

Univariate and multivariate logistic regression analyses were conducted to further investigate the association between GSD and NAFLD. GSD (OR = 1.16, 95%CI: 1.007–1.340, *P* = 0.04), and cholecystectomy (OR = 1.823, 95%CI: 1.515–2.192, *P* < 0.001) were significantly associated with the prevalence of NAFLD in the univariate analysis, and asymptomatic gallstone (OR = 0.647, 95%CI: 0.528–0.793, *P* < 0.001) was likely to be negatively associated with NAFLD. After adjustment for age, sex, BMI, blood pressure, diabetic duration, HbAlc, TC, TG and LDL-C, GSD (OR = 1.241, 95%CI: 1.036–1.488, *P* = 0.002) and cholecystectomy (OR = 1.946, 95%CI: 1.546–2.445, *P* < 0.001) were still associated with increased risk of NAFLD, while asymptomatic gallstone (OR = 0.663, 95%CI: 0.513–0.856, *P* = 0.002) was just the opposite (Table 4).

**Table 4:** Univariate and multivariate analyses for the presence of NAFLD according to GSD status

Variable	Univariate model		Multivariate model	
	OR (95%CI)	***P*** value	OR (95%CI)	***P*** value
Gallstone disease				
Control (n = 3354)	1		1	
Gallstone disease (n = 971)	1.161 (1.007–1.340)	0.04	1.241 (1.036–1.488)	0.002
Cholecystectomy				
Control	1		1	
Cholecystectomy (*n* = 538)	1.823 (1.515–2.192)	< 0.001	1.946 (1.548–2.445)	< 0.001
Gallstones				
Control	1		1	
Gallstones (*n* = 433)	0.647(0.528–0.793)	< 0.001	0.663(0.513–0.856)	0.002

The multivariate model was adjusted for age, sex, BMI, waist circumference, diabetic duration, SBP, DBP, HbA1c, TC, TG and LDL

NAFLD, nonalcoholic fatty liver disease; OR, odds ratio; CI, confidence interval

## Discussion

NAFLD and GSD are common comorbid conditions that are associated with the same risk factors, including insulin resistance, obesity, metabolic syndrome, and T2DM [15–18]. Insulin resistance is considered a pivotal feature of both NAFLD and cholesterol gallstones [19, 20]. There is increasing evidence indicating that NAFLD may be associated with the occurrence of GSD [21–24]. Here, we explored the relationship between GSD and NAFLD in patients with T2DM. However, no significant difference in the prevalence of GSD in patients with diabetes and NAFLD was observed in our study. T2DM may account for this result. According to an epidemiological study, the prevalence of GSD in people with Han Chinese ethnicity is approximately 12% [21], whereas in the current study, which also had a Han Chinese cohort, the prevalence of GSD in patients with T2DM was 22.45%. In addition, whether in the general population or in patients with T2DM, patients with NAFLD have a 2.5% higher prevalence of GSD than those without NAFLD [11]. These results indicate that diabetes is a much stronger risk factor than NAFLD for GSD. In other words, the prevalence of GSD in patients with T2DM is so high that it obscures the effects of NAFLD.

In this study, we analyzed the prevalence of asymptomatic gallstones and cholecystectomy. The relationship between asymptomatic gallstones and NAFLD remains controversial [22]. However, the correlation between cholecystectomy and NAFLD has been relatively consistent, suggesting that cholecystectomy is an independent risk factor for NAFLD [25, 26]. We also observed an increased prevalence of NAFLD in patients with T2DM and a history of cholecystectomy. Moreover, this study was the first to report that the prevalence of NAFLD was lower in patients with asymptomatic gallstones than in patients without GSD. Since the related studies were mainly cross-sectional studies, the sequential relationship between cholecystectomy and NAFLD remains unclear. Considering the similar prevalence of GSD in patients with and without NAFLD as well as the low prevalence of asymptomatic gallstones and considerably high prevalence of cholecystectomy in patients with NAFLD, we suggest that the incidence of complications of GSD (including biliary colic) in patients with diabetes and NAFLD is higher than those patients with diabetes but without NAFLD, resulting in a higher rate of cholecystectomy. Although previous studies and ours have observed that NAFLD is positively correlated with cholecystectomy, our data imply that NAFLD may be a risk factor rather than a consequence of cholecystectomy. Another piece of evidence supporting our hypothesis is that a prospective study discovered that the prevalence of NAFLD in symptomatic gallstones patients was 55% by performing intraoperative liver biopsy at the end of their cholecystectomy, which is nearly 1.7 times more than that in the general population of same ethnic observed before [27, 28]. In another word, a greater part of NAFLD occurred before cholecystectomy.

Till now, direct evidence proving the chronological sequence of GSD and NAFLD or cholecystectomy and NAFLD is lacked. Meanwhile, the exact mechanism underlying the association between NAFLD and GSD or cholecystectomy still remains unknown. Hepatic insulin resistance may create an inner link between GSD and NAFLD, which is widely accepted as an independent risk factor for both conditions [29–31]. The excess accumulation of lipids in NAFLD leads to local inflammation and aggravating insulin resistance by upregulating relevant cytokines [19, 32–34]. In turn, insulin resistance notably increases cholesterol secretion by upregulating mRNA levels of cholesterol exporters ABCG5/G8 in hepatocytes and reducing enzymes relating to bile acids synthesis like CYP7A1, CYP7B1, sterol 12 α-hydroxylase (CYP8b1) and sterol 27-hydroxylase (CYP27A1), which results in cholestasis [35–37]. Furthermore, the increasing bile acids may suppress the farnesoid X receptor (FXR) signaling in the liver and thus reducing serum FGF19 [38]. In view of the markedly protective roles that FXR signal pathway and FGF19 play in GSD [39], NAFLD impairing this pathway may account for the increasing incidence of GSD complications.

This study had several limitations. First, the lack of a nondiabetic control group may limit the generalizability of our results. Second, our findings are limited to Chinese population; therefore, this conclusion may not be generalizable to other populations in other geographical locations. Third, the cross-sectional design precluded the determination of a causative relationship. Longitudinal studies with long-term follow-up and larger sample sizes are required. Finally, both the inner mechanism of NAFLD and the underlying pathophysiology of the association between NAFLD and GSD are so complex since a lot of co-variates or mediation factors could not be excluded, distinguishing causes from effects is very difficult [40–42].

## Conclusions

In our cohort of patients with T2DM, the prevalence of GSD was not associated with NAFLD, and the higher incidence of complications of GSD was likely related to NAFLD, which is reflected in the higher proportion of cholecystectomy in NAFLD patients. NAFLD may induce the complications of GSD, leading to a higher prevalence of cholecystectomy.

## Data Availability

The datasets used and/or analyzed during the current study are available from the corresponding author on reasonable request.

## Abbreviations

NAFL(D): Nonalcoholic fatty liver (disease)
GSD: Gallstone disease
T2DM: Type 2 diabetes mellitus
NASH: Nonalcoholic steatohepatitis
BMI: Body mass index
WC: Waist circumference
FPG: Fasting plasma glucose
TG: Triglyceride
TC: Total cholesterol
HDL-C: High-density lipoprotein cholesterol
LDL-C: Low-density lipoprotein cholesterol
HbA1c: Glycosylated hemoglobin
GA: Glycosylated albumin
GGT: Gamma-glutamyl transpeptidase
ALT: Alanine aminotransferase
AST: Aspartate aminotransferase
